# Activated PPAR*γ* Targets Surface and Intracellular Signals That Inhibit the Proliferation of Lung Carcinoma Cells

**DOI:** 10.1155/2008/254108

**Published:** 2008-08-13

**Authors:** Shou Wei Han, Jesse Roman

**Affiliations:** ^1^Division of Pulmonary, Allergy and Critical Care Medicine, Department of Medicine, Emory University School of Medicine, 615 Michael Street Suite 205, Atlanta, GA 30322, USA; ^2^Atlanta VA Medical Center, Department of Medicine, 1670 Clairmont Road, Decatur, GA 30033, USA

## Abstract

Peroxisome proliferator-activated receptors (PPARs) are ligand-activated transcription factors belonging to the nuclear hormone receptor superfamily. Their discovery in the 1990s provided insights into the cellular mechanisms involved in the control of energy homeostasis, the regulation of cell differentiation, proliferation, and apoptosis, and the modulation of important biological and pathological processes related to inflammation and cancer biology, among others. Since then, PPARs have become an exciting target for the development of therapies directed at many disorders including cancer. PPARs are expressed in many tumors including lung cancer, and their function has been linked to the process of carcinogenesis. Consequently, intense research is being conducted in this area with the hope of discovering new PPAR-related therapeutic targets for the treatment of lung cancer. This review summarizes the research being conducted in this area, and focuses on the mechanisms by which a member of this family (PPAR*γ*) is believed to affect lung tumor cell biology.

## 1. INTRODUCTION

Lung carcinoma is one of the most common malignant tumors in the world, and is the leading cause of carcinoma death in USA [1]. Primary malignant cancers of the lung are classified into small cell lung cancer (SCLC) and nonsmall cell lung cancer (NSCLC). NSCLC accounts for 80% of malignant lung cancer, and SCLC constitutes the remainder [2]. Based on the cellular phenotype, NSCLC is further subdivided into squamous cell carcinoma, adenocarcinoma, and large cell carcinomas [3]. Despite advances in understanding the mechanisms involved in carcinogenesis, the development of new surgical procedures, and the use of new radio and chemotherapeutic protocols, the five-year survival rate for lung cancer patients is poor and remains less than 15% [1]. This underscores the desperate need for novel strategies for early detection, prevention, and treatment of this malignancy.

Since their discovery in 1990, peroxisome proliferator-activated receptors (also known as PPARs) have captured the attention of investigators interested in learning about the intracellular pathways that control signal transduction and gene transcription. PPARs were originally cloned in an attempt to identify the molecular mediators of peroxisome proliferation in the liver of rodents. Today, PPARs are recognized as versatile members of the ligand-activated nuclear hormone receptor superfamily of transcription factors that includes receptors for steroids, thyroid hormone, retinoic acid, and vitamin D, among others [4]. PPARs are considered to play key roles in diverse physiological processes ranging from lipid metabolism to inflammation, and have been implicated in diseases such as cancer, atherosclerosis, and diabetes [4, 5]. Although information about the function of PPARs in lung is scarce, data implicating these molecules in key processes in lung biology are rapidly emerging.

Three subtypes of PPARs have been identified and cloned: PPAR*α*, PPAR*β*/*δ*, and PPAR*γ*. These subtypes are distinguished by their tissue distribution, and to a lesser degree, by their ligand specificity. PPAR*α* has been implicated in hepatocellular carcinoma in rodents, whereas activation of PAPR*β*/*δ* promotes human lung carcinoma cell proliferation through PI3-Kinase/Akt activation [6]. However, of the three PPARs identified to date, PPAR*γ* represents the most promising target in view of the many reports implicating this molecule in lung carcinoma cell growth both in vitro and in vivo. This review focuses on PPAR*γ*, its role in lung carcinogenesis, and the potential therapeutic role of PPAR*γ* agonists in lung cancer.

## 2. ELUCIDATING THE FUNCTION OF PPAR*γ*

PPAR*γ* was discovered based on its similarity to PPAR*α*, and it is the most intensively studied ligand-dependent transcriptional regulator. By utilizing three different promoters, a single PPAR*γ* gene encodes three isoforms, namely, PPAR*γ*1, PPAR*γ*2, and PPAR*γ*3 [7]. Analysis of PPAR*γ*1 and *γ*3 transcripts revealed that they both translate into the same PPAR*γ*1 protein [8]. PPAR*γ*2 protein contains additional 30 amino acids at its N-terminus compared to PPAR*γ*1. PPAR*γ* is highly expressed in adipose tissue, and it is a master regulator of adipocyte differentiation [9, 10]. In addition to its role in adipogenesis, PPAR*γ* serves as an important transcriptional regulator of glucose and lipid metabolism, and it has been implicated in the regulation of insulin sensitivity, atherosclerosis, and inflammation [11, 12]. PPAR*γ* is also expressed in multiple other tissues such as breast, colon, lung, ovary, prostate, and thyroid, where it was demonstrated to regulate cellular proliferation, differentiation, and apoptosis [13, 14]. As will be discussed later, PPAR*γ* activation inhibits tumor progression in NSCLC [15, 16]. Several leukocyte populations, including monocytes/macrophages, lymphocytes, and dendritic cells, have also been shown to express PPAR*γ* suggesting a role for this molecule in the regulation of immune responses [17]. PPAR*γ* has been described as a negative regulator of macrophage function since its activation suppresses the production of inflammatory cytokines, chemokines, metalloproteases, and nitric oxide [18, 19]. These PPAR*γ*-mediated anti-inflammatory effects are not restricted to monocytes, as the treatment with PPAR*γ* agonists results in inhibition of cytokine/chemokine production in several epithelial and stromal cells [15].

Several natural and synthetic compounds have been identified as activators of PPAR*γ*. The insulin-sensitizing antidiabetic drugs known as thiazolidinediones (TZDs) were the first compounds identified as PPAR*γ* agonists [20]. The TZDs' rosiglitazone and pioglitazone are currently in clinical use for the treatment of type II diabetes, while troglitazone was withdrawn from clinical use because it was linked to idiosyncratic liver toxicity [21]. Other non-TZD synthetic ligands include certain nonsteroidal anti-inflammatory drugs such as isoxazolidinone JTT-501 [22] and tyrosine-based GW7845 [23]. Naturally occurring compounds that activate PPAR*γ* include long-chain polyunsaturated fatty acids which are found in fish oil (e.g., n-3-PUFA and n-6-PUFA), eicosanoids (e.g., 15d-PGJ2), lipid hydroperoxides (e.g., 9(s)-HODE and 13(s)-HODE), as well as linoleic acid, 15-deoxy-Δ^12,14^ prostaglandin J_2_ (15d-PGJ_2_), 12/15 lipoxygenase products of 15-hydroxyeicosatetraenoic acid (15-HETE), and 13-hydroxyoctadecadienoic acid [24–26]. In addition, compounds from several medicinal plants such as Saurufuran A from *Saururus chinensis* [27], flavonoids like chrysin and kaempferol [28], phenolic compounds from *Glycyrrhiza uralensis* [29], and curcumin from *Curcumin longa* [30, 31] are also shown to activate PPAR*γ*.

The synthetic ligands and some natural ligands have been used to elucidate the role of PPAR*γ* in cellular functions both in vitro and in vivo. However, several caveats should be taken into consideration when interpreting such studies. First, the natural ligands that regulate PPARs in vivohave not been completely elucidated. Second, not all PPAR*γ* ligands exert their effects through PPAR*γ* since there is strong evidence for the activation of PPAR*γ*-independent signals, particularly with the natural ligand 15d-PGJ_2_. Third, high-affinity ligands for PPAR*γ* (e.g., TZDs) may exert partial agonist/antagonist activity [32]. The latter might be due to the fact that individual TZDs induce different PPAR*γ* conformations that influence the recruitment of different coactivator/corepressor molecules. Thus, the activity of the PPAR*γ* transcriptional complex is influenced by the context of a given gene and its promoter, and by the relative availability of pertinent coactivator/corepressor molecules in the cell or tissue of interest.

## 3. PPAR*γ* IN LUNG CANCER

**Figure 1:**
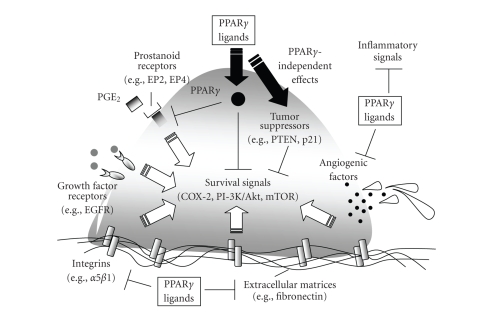
*Anticancer actions of PPAR*γ* ligands*. In addition to genetic abnormalities, lung carcinoma cells receive mitogenic and antiapoptotic signals that promote their progression and metastasis through the activation of key intracellular pathways (e.g., COX-2, Akt, and mTOR). Lung carcinoma cells recognize these signals via diverse receptors for growth factors (e.g., EGFR), prostanoids (e.g., EP2 and EP4), and extracellular matrices (e.g., integrins), among others. In addition, angiogenic factors assist in the vascularization of tumors, while inflammatory signals further promote tumor progression. PPAR*γ* ligands inhibit tumor growth in animal models, but the mechanisms responsible for these effects appear to be multidimensional. In vitro studies reveal that PPAR*γ* ligands affect tumors by inhibiting the expression of key prostanoid and integrin receptors, by reducing the expression of fibronectin, a matrix glycoprotein that stimulates tumor cell proliferation, and by inhibiting the production of angiogenic and inflammatory signals. In addition, PPAR*γ* ligands increase the expression and/or activity of tumor suppressors like PTEN and p21. Although many of these anticancer effects are mediated through PPAR*γ*, others appear to be independent of this nuclear transcription factor (e.g., via targeting TSC2, AMPK, and ROS production and ERK activation, and interacting with CRE, AP-1).

Among the three subtypes, the role of PPAR*γ* has been investigated the most in lung carcinogenesis. PPAR*γ* is expressed in many cancers including colon, breast, and prostate cancers, and with few exceptions, PPAR*γ* ligands are antiproliferative in these cancers. Similarly, PPAR*γ* is expressed in SCLC and NSCLC [33]. Furthermore, PPAR*γ* ligands induce growth arrest and promote changes associated with differentiation as well as apoptosis in a variety of lung carcinoma cell lines although most of the knowledge available in this area has been generated in NSCLC [34, 35]. The exact mechanisms linking modulation of PPAR*γ* with cancer growth inhibition remain unclear. However, current evidence suggests that PPAR*γ* ligands affect a number of mechanisms including regulation of the intracellular machinery involved in cell signaling and cell cycle control, inhibition of mitogenic factors and tumor promoters, prevention of tumor cell recognition of extracellular mitogenic signals, breakdown of nicotine-induced cell survival, and modulation of the expression of angiogenic factors needed for the development of the vascular networks that supply tumor cell (see Figure 1). These mechanisms are discussed below as they relate to the action of PPAR*γ* ligands in lung cancer.

Several studies demonstrate that PPAR*γ* ligands affect cell cycle control in tumor cells. For example, PPAR*γ* ligands have been found to inhibit the growth of A549 adenocarcinoma cells due to G0/G1 cell cycle arrest through the upregulation of mitogen-activated protein kinases ERK1/2 and the downregulation of G1 cyclins D and E [15]. Troglitazone inhibits NSCLC proliferation in part by stimulating the expression of the GADD 153 (for growth arrest and DNA damage-inducible gene-153) [36]. Also, troglitazone was found to induce apoptosis in NCI-H23 cells via a mitochondrial pathway through the activation of ERK1/2 [37]. Others have shown similar results using CRL-202 cells, and further demonstrated that troglitazone downregulated the expression of the antiapoptotic molecules Bcl-w and Bcl-2, as well as decreasing the activity of SAPK/JNK [38]. PPAR*γ* ligands also induce the expression of death receptor 5 (DR5) and increase DR5 distribution at the cell surface in addition to reducing c-FLIP levels in human lung cancer cells. These agents cooperated with TRAIL to enhance apoptosis in human lung carcinoma cells [39]. One recent report found that PPAR*γ* ligands 1-[(trans-methylimino-N-oxy)-6-(2-morpholinoethoxy)-3-phenyl-(1H-indene-2-carboxylic acid ethyl ester (KR-62980)] and rosiglitazone induce NSCLC apoptotic cell death mainly through PPAR*γ*-dependent reactive oxygen species formation via increased expression of proline oxidase, a redox enzyme expressed in mitochondria [35].

Tumor suppressor genes are also affected by PPAR*γ* ligands. For example, PGJ_2_ and ciglitazone stimulated the expression of p21 mRNA and protein expression in NSCLC, and this coincided with a reduction in cyclin D1 mRNA expression [40]. Of note, p21 antisense oligonucleotides significantly blocked lung carcinoma cell growth inhibition observed with PPAR*γ* ligands, thereby establishing an important role for p21 in this process. These findings are consistent with those of others showing that the proliferation of A549 cells injected subcutaneously into nude mice was inhibited significantly by treatment with ciglitazone, and this coincided with increased expression of PPAR*γ* and p21 and with downregulation of cyclin D1 [41]. A connection between another tumor suppressor gene (p53) and PPAR*γ* ligands has also been demonstrated by showing that 15-deoxy-PGJ_2_, together with docetaxel, stimulates apoptosis in NSCLC through inhibition of Bcl2 and cyclin D1 and overexpression of caspases and p53 [34].

Other reports implicate alterations in the mammalian target of rapamycin (mTOR) signaling pathway in the antitumor effects of PPAR*γ* ligands. Rosiglitazone, for example, was reported to reduce the phosphorylation of Akt, an upstream positive modulator of mTOR, and to increase PTEN, a negative modulator of mTOR, in NSCLC H1792 and H1838 cells; this resulted in inhibition of cell proliferation [42]. Although the effects of rosiglitazone on Akt and PTEN were blocked by the selective PPAR*γ* antagonist GW9662 and restored by transient overexpression of PPAR*γ*, cell growth was not entirely restored suggesting the involvement of additional PPAR*γ*-independent mechanisms of action. Further work revealed that rosiglitazone increased the phosphorylation of AMPK*α*, a target of LKB1, and TSC2, another potential tumor suppressor and upstream downregulator of mTOR. The latter pathway was independent of PPAR*γ* since GW9662 and PPAR*γ* siRNA did not affect it [42, 43]; others have shown similar increases in PTEN expression induced by rosiglitazone [44].

More recently, we found that rosiglitazone and dietary compounds such as fish oil (which contain certain kinds of fatty acids like *ω*3 and *ω*6 polyunsaturated fatty acids known to work as PPAR*γ* ligands) inhibit integrin-linked kinase (ILK) expression through PPAR*γ* signaling and the recruitment of a PPAR*γ* coactivator, PGC-1*α* (Han et al., unpublished data). ILK is a unique intracellular adaptor and kinase that links cell-adhesion receptors, integrins, and growth factors to the actin cytoskeleton and to a range of signaling pathways that are implicated in the regulation of anchorage-dependent tumor cell growth/survival, cell cycle progression, invasion and migration, and tumor angiogenesis [45]. This effect was associated with activation of p38 MAPK followed by induction of transcription factor AP-2*α*. In turn, this resulted in inhibition of NSCLC cell proliferation (Han et al., unpublished data).

Several studies suggest that PPAR*γ* ligands also exert antitumor effects by blocking access to mitogenic agents such as PGE_2_, a major cyclooxygenase metabolite that plays important roles in tumor biology. The functions of PGE_2_ are mediated through one or more of its receptors: EP1, EP2, EP3, and EP4 [46]. Human NSCLC cell lines express EP2 receptors, among other EP receptors, and the inhibition of cell growth by PPAR*γ* ligands like GW1929, PGJ_2_, ciglitazone, troglitazone, and rosiglitazone is associated with a significant decrease in EP2 mRNA and protein expression. Notably, the inhibitory effects of rosiglitazone and ciglitazone, but not PGJ_2_, were reversed by a specific PPAR*γ* antagonist GW9662, suggesting the involvement of PPAR*γ*-dependent and PPAR*γ*-independent mechanisms [46]. Also, a recent study showed that ciglitazone suppressed cyclooxygenase-2 (COX-2) mRNA expression and COX-2 promoter activity, while upregulating peroxisome proliferators' response element (PPRE) promoter activity in NSCLC cells, further suggesting a negative modulator role for PPAR*γ* ligands in the COX-2/PGE_2_ pathway in NSCLC [47].

Nicotine, a major component of tobacco, stimulates NSCLC cell proliferation through nicotinic acetylcholine receptor- (nAChR-) mediated signals. A recent case-control study of 500 incident lung cancer cases and 517 age and sex frequency-matched cancer-free controls suggested that PPAR*γ* polymorphisms in Chinese smokers may contribute to the etiology of lung cancer [48]. Monocytes and monocyte-derived macrophages from healthy smokers showed increased PPAR*γ* expression as compared to those from healthy nonsmokers, which were reproduced by nicotine in vitro [49]. Interestingly, concomitant administration of PPAR*γ* agonists can effectively attenuate the effects of nicotine on alveolar type II cells [50]. We recently found that rosiglitazone reduced nicotine-induced NSCLC cell growth through downregulation of *α*7 nAChR-dependent signals including ERK and p38 MAPK; this effect appeared to be PPAR*γ*-independent (Han et al., unpublished data). If confirmed, this may unveil a novel mechanism by which rosiglitazone inhibits human lung carcinoma cell growth.

Other studies suggest that PPAR*γ* ligands might prevent the interaction of tumor cells with their surrounding stromata, thereby interfering with host-derived and tumor-derived factors and mitogenic and prosurvival effects. An example of this is fibronectin, a matrix glycoprotein residing in the lung stroma that is increased in most, if not all, chronic forms of lung disease [51]. This is true for tobacco-related lung disorders and fibrotic disorders—all associated with increased incidence of lung cancer [52]. Several studies suggest that fibronectin serves as a mitogen and survival factor for NSCLC [53], and fibronectin has been recently shown to stimulate tumor cell expression of matrix metalloproteinases, proteases implicated in metastatic disease [54]. These observations support the idea that tumor cell interactions with fibronectin through surface integrin receptors are advantageous for tumors since they stimulate proliferation, survival, and metastases [53]. This idea remains to be proven in vivo, but if found to be true, this might unveil a new target for anticancer strategies. In this regard, PPAR*γ* ligands were shown to inhibit fibronectin expression in NSCLC cells by inhibiting transcription factors involved in regulation of fibronectin gene expression [55]. PPAR*γ* ligands (rosiglitazone and GW1929, but not PGJ_2_) have been also recently reported to inhibit the expression of the gene encoding for the *α*5 integrin subunit resulting in reduced expression of the integrin *α*5*β*1, a fibronectin receptor that mediates fibronectin's mitogenic effects in NSCLC cells and nontumor lung cells [56]. Thus, by inhibiting the expression of fibronectin and its integrin *α*5*β*1, PPAR*γ* ligands might reduce tumor cell recognition of fibronectin with consequent changes in cell proliferation and apoptosis.

PPAR*γ* might also regulate the generation of the complex vascular network that supplies tumor cells. This idea is supported by studies showing a reduction in blood vessel density in the lung tumors generated by the injection of A549 cells into the flanks of SCID mice treated with PPAR*γ* ligands [57]. In in vitro studies, the treatment of A549 cells with troglitazone or their transient transfection with a constitutively active PPAR*γ* construct blocked the production of angiogenic molecules such as ELR+CXC chemokines IL-8 (CXC-8), ENA-78 (CXCL5), and Gro-alpha (CXCL1) [57]. Furthermore, PPAR*γ* activation inhibited NF-*κ*B, a transcription factor known to regulate the expression of many of the proangiogenic factors mentioned above. Similarly, rosiglitazone was shown to inhibit mouse lung tumor cell growth and metastasis in vivo through direct and indirect antiangiogenic effects [16]. It is important to note that PPAR*γ* signaling has also been associated with tumor promoter activities in some tumor cells such as colon and breast, and that this effect was linked to increased beta-catenin and c-Myc expression [58, 59] (Table 1). These findings need to be confirmed and tested in other tumors. However, these data suggest that activation of specific PPAR*γ*-related pathways may differ depending upon the cells and tumors examined. More than one pathway was involved in the effect of PPAR*γ* ligands in one cell line which was not observed in others. Internal genetic variations and other factors may be responsible for these outcomes, and these need to be explored further followed by confirmation in in vivo models of cancer.

## 4. IMPLICATIONS FOR THERAPY

The studies mentioned above suggest that PPARs are involved in lung cancer cell biology. However, their roles remain uncertain and much needs to be learned before they are targeted for therapeutic intervention, especially when considering PPAR*γ*. Nevertheless, activation of PPAR*γ* is strongly associated with decreased lung carcinoma cell proliferation both in vitro and in vivo. Furthermore, in primary NSCLC, the expression of PPAR*γ* has been correlated with tumor histological type and grade, and decreased PPAR*γ* expression was correlated with poor prognosis [60]. Because of this and the fact that synthetic agonists of PPAR*γ* with good safety profiles are currently in use in the clinical arena, PPAR*γ* has emerged as a reasonable target for the development of novel antilung cancer therapies. Synthetic and natural PPAR*γ* activators might be useful as well. For example, arachidonic acid inhibits the growth of A549 cells, and this effect is blocked by the synthetic PPAR*γ* inhibitor GW9662 [61]. MK886, a 5-lipoxygenase activating protein-directed inhibitor, stimulates apoptosis and reduces the growth of A549 cells through activation of PPAR*γ* [62]. These and related drugs can be used alone or in combination with other drugs for synergistic effects. This was observed when using low doses of MK886 in combination with ciglitazone and 13-cis-retinoic acid on A549 and H1299 cells [62]. Also, dramatic synergistic anticancer effects have been reported for lovastatin (an HMG-CoA reductase inhibitor) and the PPAR*γ* ligand troglitazone in several cell lines including lung cancer cells [63]. An enhancement of the antitumor effects of gefitinib by rosiglitazone on A549 cell growth has been recently noted, suggesting that combination strategies using selective nuclear receptor activators in conjunction with epidermal growth factor receptor inhibitors might be effective [64].

A recent study demonstrated that combining the PPAR*γ* ligand rosiglitazone with carboplatin dramatically reduced lung tumor growth in vivo [65]. More tantalizing data were derived from retrospective analysis demonstrating that thiazolidinedione (TZD) use was associated with reduced risk of lung cancer. This study revealed 33% reduction in lung cancer risk among thiazolidinedione users as compared to the nonusers after adjusting other variables [66]. Interestingly, similar risk reduction was not observed for colorectal and prostate cancers [66].

Despite the above, enthusiasm for the use of PPAR*γ* ligands as anticancer agents should be tempered by the fact that PPAR*γ* ligands stimulated PPAR*γ* transactivation in lung adenocarcinoma cell lines, while few to no effects were noted in squamous cell or large cell carcinomas. Also, it is important that we better define PPAR*γ*-independent pathway to avoid unforeseen events and to identify new targets for intervention [64, 67] (Table 2). Furthermore, a novel splice variant of human PPAR*γ*1 which is expressed strongly in tumor tissues of primary human lung SCC has been recently identified. This splice variant exhibits dominant-negative properties in human lung tumor cells, and its overexpression renders transfected cells more resistant to chemotherapeutic drug- and chemical-induced cell death [68]. This suggests that the decreased drug sensitivity of PPAR*γ*1-expressing cells may be associated with increased tumor aggressiveness and poor clinical prognosis in patients. Thus, a better understanding of the mechanisms of action of activated PPARs in tumors (and host cells) is required since the dissection of these pathways might unveil better targets for therapy. Nevertheless, the data available to date regarding PPAR*γ* are promising and justify engaging in clinical studies to determine the true role of PPAR*γ* ligands in lung cancer, while further work should be performed to identify more selective and effective strategies.

## 5. CONCLUSION

In summary, although its exact role in controlling lung tumor growth and apoptosis remains undefined, PPAR*γ* has been implicated both as a tumor suppressor (in most cases) and as a tumor promoter (in rare cases). Hence, targeting this receptor for therapeutic purposes while minimizing side effects represents a great challenge. Nevertheless, it is clear that selective PPAR*γ* modulation of desired gene sets can be achieved by targeting corepressor interactions, separating transactivation from transrepression, and favoring specific subsets of coactivators. Although the exact mechanisms mediating this effect remain incompletely elucidated, data available to date regarding this member of the PPAR family are promising and justify engaging in prospective, randomized clinical studies to determine the true role of PPAR*γ* ligands in lung cancer biology.

## Figures and Tables

**Table 1:** PPAR*γ*-dependent signals in mediating the effects of PPAR*γ* ligands.

(1) PPAR*γ* ligands inhibit cancer **cell growth** and induce apoptosis via:
↓ PGE_2_ **receptors (e.g., EP2 and EP4)
↑ Tumor suppressors (e.g., PTEN, p21)
↓ Inflammatory factors (e.g., NF-*κ*B, MCP-1, COX-2)
↓ Angiogenic **factors (e.g., VEGF)
↓ Survival factors (e.g., PI3-K/Akt, mTOR)
↑↓ Other kinase signals (e.g., ERK, p38 MAPK)
↓ Growth **factor **receptors (e.g., EGF-R, PDGF-R)
↓ Extracellular **matrices (e.g., fibronectin,**MMP-9)
↓ Integrin receptors (e.g., *α*5*β*1)
↑↓ Others (e.g., cytokines (e.g., IL-13, IL-21, TGF-*β*1) and chemokines (e.g., MIP-1*β*))

**Table 2:** PPAR*γ*-independent signals triggered by PPAR*γ* ligands.

(2) PPAR*γ* ligands stimulate cancer **cell growth** and reduce **apoptosis via:
↑ Wnt signaling and oncogenes (e.g., cyclin D1, *β*-catenin, **c-myc)
↑ Tumor suppressors (e.g., LKB1, AMPK, TSC2)
↑ ROS production and ERK activation **(note that this also occurs in PPAR*γ*-dependent pathways)
↓ Effects on transcription factors (e.g., AP-1, NF-*κ*B, Smads, Sp1, CRE)
